# Assessment of a Low-Cost Ultrasound Pericardiocentesis Model

**DOI:** 10.1155/2013/376415

**Published:** 2013-10-29

**Authors:** Marco Campo dell'Orto, Dorothea Hempel, Agnieszka Starzetz, Armin Seibel, Ulf Hannemann, Felix Walcher, Raoul Breitkreutz

**Affiliations:** ^1^Abteilung Kardiologie, Kerckhoff Klinik Bad Nauheim, 61231 Bad Nauheim, Germany; ^2^Frankfurter Institut für Notfallmedizin und Simulationstraining, Fachbereich Medizin der Johann Wolfgang Goethe-Universität, Klinikum der Johann Wolfgang Goethe-Universität, 60528 Frankfurt am Main, Germany; ^3^II. Medizinische Klinik und Poliklinik, Universitätsmedizin Mainz, 55131 Mainz, Germany; ^4^Klinik für Anästhesiologie, Intensiv- und Notfallmedizin, Diakonie Klinikum Jung Stilling, 57074 Siegen, Germany; ^5^Zentrale Notaufnahme, Klinikum Darmstadt, 64283 Darmstadt, Germany; ^6^Klinik für Unfall-, Hand- und Wiederherstellungschirurgie, Klinikum der Johann Wolfgang Goethe-Universität, 60590 Frankfurt am Main, Germany; ^7^Zentrale Notaufnahme, Klinikum Frankfurt Höchst, 65929 Frankfurt am Main, Germany

## Abstract

*Introduction*. The use of ultrasound during resuscitation is emphasized in the latest European resuscitation council guidelines of 2013 to identify treatable conditions such as pericardial tamponade. The recommended standard treatment of tamponade in various guidelines is pericardiocentesis. As ultrasound guidance lowers the complication rates and increases the patient's safety, pericardiocentesis should be performed under ultrasound guidance. Acute care physicians actually need to train emergency pericardiocentesis. *Methods*. We describe in detail a pericardiocentesis ultrasound model, using materials at a cost of about 60 euros. During training courses of focused echocardiography *n* = 67, participants tested the phantom and completed a 16-item questionnaire, assessing the model using a visual analogue scale (VAS). *Results*. Eleven of fourteen questions were answered with a mean VAS score higher than 60% and thus regarded as showing the strengths of the model. Unrealistically outer appearance and heart shape were rated as weakness of the model. A total mean VAS score of all questions of 63% showed that participants gained confidence for further interventions. *Conclusions*. Our low-cost pericardiocentesis model, which can be easily constructed, may serve as an effective training tool of ultrasound-guided pericardiocentesis for acute and critical care physicians.

## 1. Introduction

Pericardial tamponade is a potentially life-threatening condition and can lead to cardiac arrest. The latest ERC guidelines of 2010 emphasize the use of ultrasound during resuscitation in order to detect reversible causes such as pericardial tamponade to provide early and targeted treatment [1]. It has been shown that echocardiography can be integrated into peri-resuscitation care in an ALS-conform fashion [2]. Thus, acute care physicians will encounter patient scenarios where no specialist is available, but a treatable condition needing immediate intervention is detected. There are several conflicts. Ultrasound now can detect reversible conditions such as tamponade. Thus, emergency or critical care physicians face the problem for treating these conditions, even without experience due to a lack of training in ultrasound-guided pericardiocentesis, because training phantoms are not readily available. Also, the required equipment needed to perform pericardiocentesis is not available in all ambulances or emergency rooms.

The skill for performing a focused echocardiography exam can be acquired in brief training courses [3]. The recommended standard treatment of tamponade in various guidelines is pericardiocentesis. It has been shown that pericardiocentesis can safely be performed under echocardiographic guidance with success rates as high as 100% [4–6]. Most physicians as well as specialists have not been trained to perform pericardiocentesis during their residency and do not feel comfortable while performing it. Several ultrasound models or phantoms for teaching and training ultrasound-guided procedures, such as venous puncture, have been developed [7, 8]. For the training of pericardiocentesis, both the cadavers and pericardiocentesis training models are available [9]. These models have been shown to facilitate practicing the skills needed to perform pericardiocentesis.

However, most of the available models are expensive (i.e., the “Blue phantom” *transthoracic echocardiography and pericardiocentesis ultrasound training* model starting at 15.000 USD [10]) and cannot be afforded by many institutions, especially in low-income countries. A low-cost pericardiocentesis phantom has been published during our project timeline, but there was no systematic evaluation of its features and its effectiveness [11].

We aimed to (1) construct a low-cost pericardiocentesis phantom that can be rebuilt easily and to (2) assess its effectiveness.

## 2. Methods

### 2.1. The Design of the Pericardiocentesis Model

**Figure 1:**
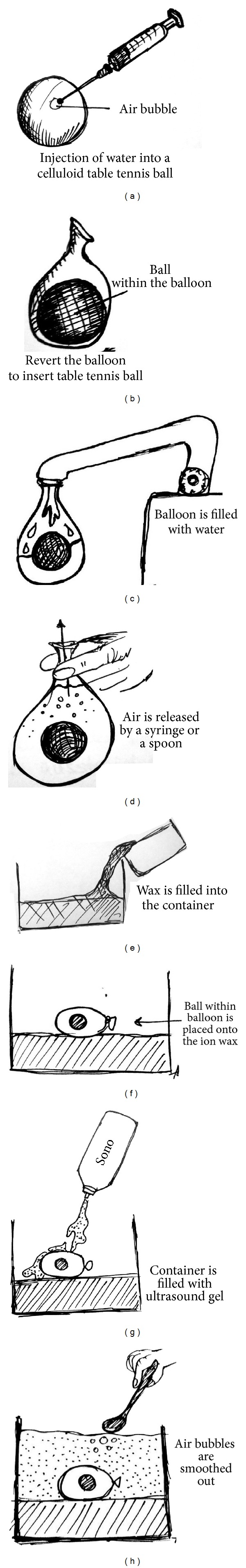
The construction of the pericardiocentesis phantom. (a) A celluloid table tennis ball is filled with water. (b) The ball is placed onto a balloon filled with ink-colored water. (c) The balloon is filled with water. (d) The balloon is dereated and knotted. (e) The container is filled with gel wax. (f) The balloon is placed onto the gel wax. (g) The container is filled with ultrasound gel. (h) All air bubbles are smoothed using a syringe or a spoon.

**Figure 2:**
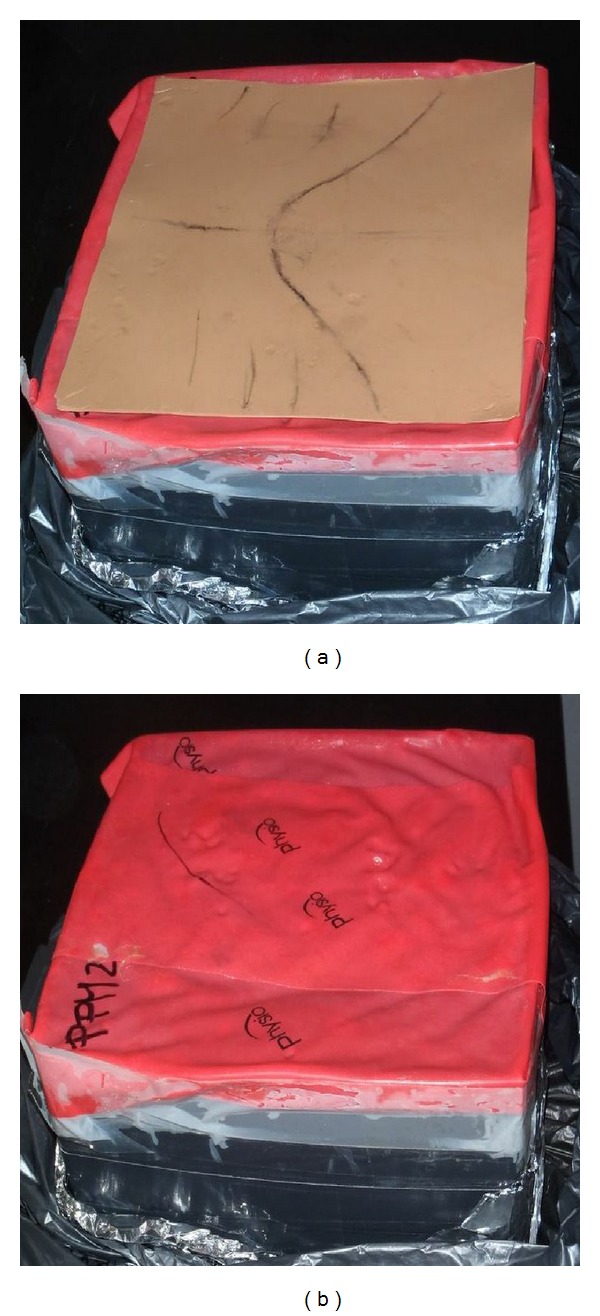
Two types of skin can be used: (a) silicone skin or (b) Thera-Band.

A square plastic container was constructed using PCV-planks (22 × 22 × 14 cm, thickness 0.3 cm), filled with gel wax, which was heated until liquid (about 80–100°C), and then filled into the container (Figure 1). The gel wax served as a stable platform within the phantom. For the heart model, a celluloid table tennis ball was filled with water using a syringe until no air was left inside. This water-filled celluloid ball was to mimic a ventricle. The ball was then put into a balloon, which was filled with red ink-colored water until it was deaerated completely and knotted. This balloon was placed onto the solidified gel wax. The container was then filled with ultrasound gel to the brim, and all the air bubbles inside the ultrasound gel were smoothed out using a spoon or a syringe (Figures 1(a)–1(h)). Finally, the model was covered by silicone skin or Thera-Band, a rubber band usually used for gymnastic exercises (Figures 2(a) and 2(b)). These were to mimic the skin piercing. All materials needed for the construction of the model cost approximately 60 euros in total, and the preparation can be completed within 2 hours. The specific materials we used and their prices in common stores are listed in Table 1. Instead of the plastic container used in our model, any square container withstanding heat can be used; the most expensive part of the model was the gel wax which also can be replaced by gelatine to reduce further costs.

### 2.2. Ultrasound Equipment and Ultrasound-Guided Pericardiocentesis

**Figure 3:**
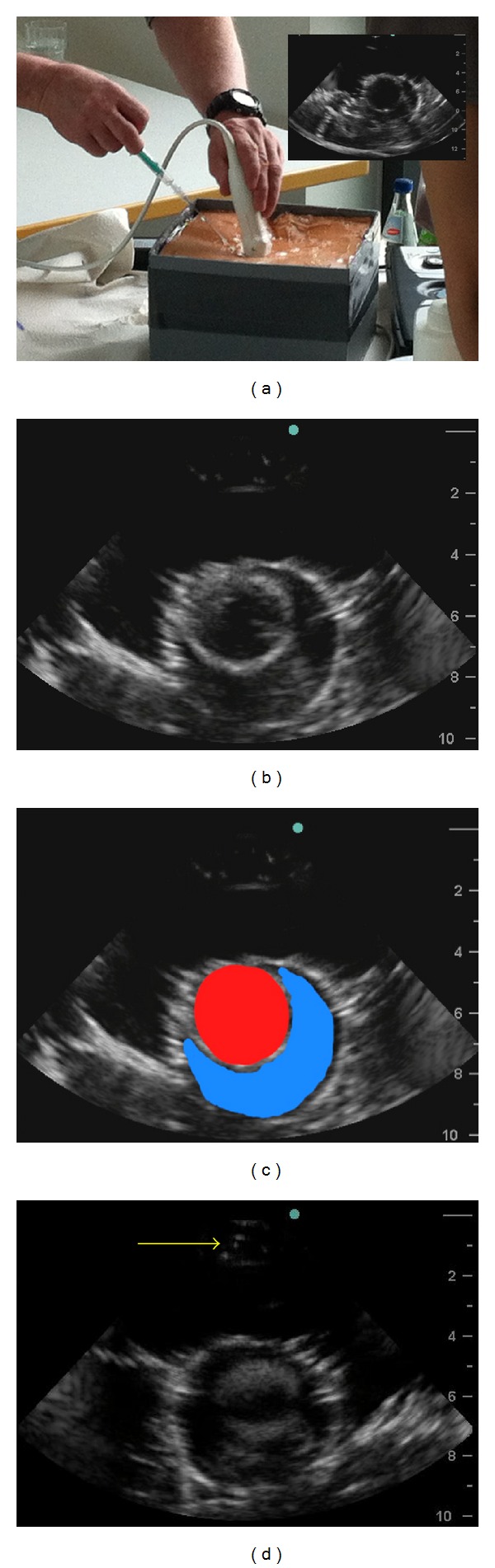
A participant is pericardiocentesis (a), the corresponding image is shown in (b), the left ventricle is colored in red, the effusion is colored in blue (c), and visualization of the needle is shown by yellow arrow (d).

**Figure 4:**
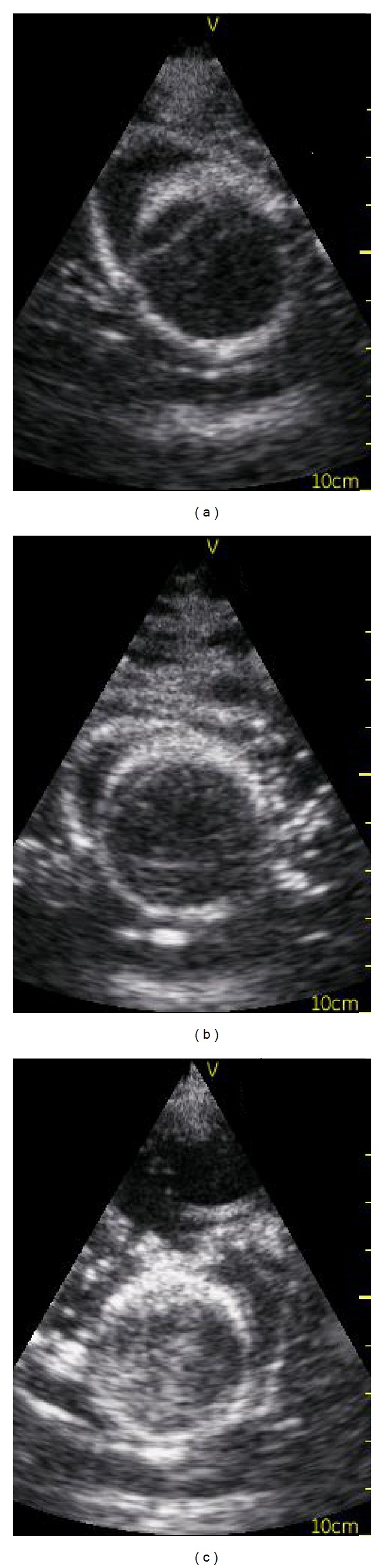
(a) before the pericardiocentesis model, (b) after puncture and aspiration of fluid, and (c) after 60 punctures and four weeks of storage; at this stage, the balloon needs to be replaced and refilled.

We used a mobile ultrasound device (Vscan, GE healthcare) for the purpose of the study. Study participants were asked to place the probe onto the model, simulating both the parasternal long- and short-axis views. Structures identified on the ultrasound image were skin, surrounding tissue, pericardium, pericardial effusion, and the heart model (left ventricle) (Figures 3(b)–3(d)). For cannulation, an 18 G needle with a 5 or 10 mL syringe attached was used. The needle was visualized during the entire procedure and advanced until seen inside the pericardium (Figure 3(d)). A reddish fluid could be aspirated if the needle was correctly placed inside the pericardium. After the tap, a decrease in the pericardial effusion volume could be seen (Figures 4(a) and 4(b)).

### 2.3. The Study Participants

The study participants were recruited during focused echocardiography in life support training courses (FEEL) organized by http://www.sonoabcd.org/. These courses, opened to physicians of all specialties, are part of a postgraduate training programme in the emergency ultrasound of Germany, Austria, and Switzerland and were certified by the German Society of Ultrasound in Medicine (DEGUM).

As a control group, medical students without prior exposure to ultrasound or pericardiocentesis were recruited for the study. All subjects participated voluntarily and granted verbal consent to allow collecting the data anonymously and to be processed and published. This trial is a part of a series of studies in emergency ultrasound from our working group and approved by the institutional review board, as stated in previous reports [2].

Participants were asked to fill out a 16-item questionnaire with a visual analogue scale (VAS) directly after using the pericardiocentesis model. Each question was accompanied by a single, horizontal 16 cm line without numbers. Participants could mark the line in between two extremes (example question: “How well can the heart be recognized in the phantom?” ranging from “very bad” to “very good”). The marks were measured to an accuracy of 1 mm, and those measurements were converted into percent values (Table 2). The questions were related to visualization of heart, pericardium and effusion, visualization of the needle, sense of puncture through the pericardium, how close they felt the model came to reality, and if they felt that the model increased their self-assurance in regard to ultrasound-guided pericardiocentesis (Table 2).

Additionally, participants were asked if they had performed any kind of puncture, no punctures before, or if they had distinct experience in pericardiocentesis. Question 16 asked all participants about their profession and their specialty.

Three groups of participants were defined according to the level of previous experience with pericardiocentesis or punctures. Group 1 consisted of participants who had no experience with punctures of any kind (*n* = 41). Group 2 was formed of participants who had not performed any pericardiocentesis before but had experience in performing other kinds of punctures (i.e., neuromuscular blocks and central lines) (*n* = 17). Group 3 consisted of participants who marked that they had performed pericardiocentesis before (*n* = 9).

Statistical analysis was performed using GraphPad Prism 5, La Jolla. Only descriptive statistics (mean, median, and standard deviation) were calculated. The scores of the three groups were compared using the Mann-Whitney *U* test in order to identify the differences in ratings between the groups.

A percentage value above 60% was defined to represent the strengths of the phantom and the value below 60% to represent the weaknesses. The liberal 60% cutoff value was chosen, as the model was not created to represent reality as best as possible but to simulate the act of puncture using a readily available phantom.

## 3. Results

The model was constructed several times by a fellow (AS) and was assembled in a mean time of about 2 hours. A single phantom was used for more than 60 punctures without being destroyed. The ultrasound appearance of the model after more than 60 punctures and 4 weeks of storage at room temperature is shown in Figure 4(c). Only the balloon with the enclosed celluloid ball had to be replaced so that the model could be used for further training. The first model was constructed in 2011 and showed no signs of moulding or decay even after more than 24 months of storage at room temperature.

**Figure 5:**
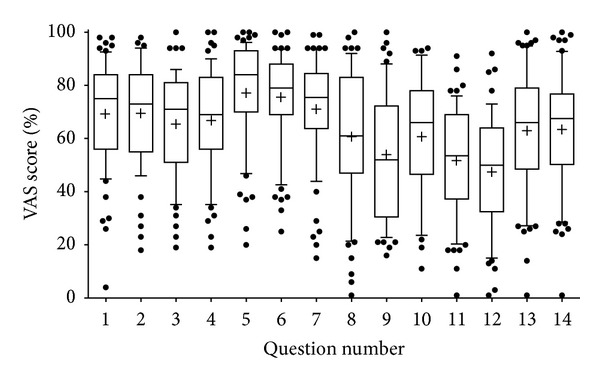
Results of pooled VAS score per question. Each number of the *x*-axis represents a question of Table 2. Data were pooled, because there was no difference in the evaluation results between the three test groups (participants *n* = 67).

A total of *n* = 67 participants (specialties: 40 anaesthesia, 13 internal medicine, 3 surgery, 4 students, and 7 physicians without specialisation) used the pericardiocentesis model and filled out a questionnaire during two ultrasound courses in April and June 2013. As specified above, three groups were defined according to the level of experience regarding pericardiocentesis. There was no statistically significant difference in the answer score to any of the 16 questions between the 3 groups. Therefore, data were pooled and presented as box plots (Figure 5). Mean scores for questions 1 to 14 ranged from 48% to 78%. The 60% cutoff value was surpassed in 11/14 questions. The weaknesses of the phantom were questions 9, 11, and 12, showing that the model was regarded as not realistic by most participants, and that the decrease in fluid could not be followed well. The highest scores (78% ± 19, mean ± SD, and 76% ± 18, resp.) were reached for questions regarding the visibility of the effusion and its discrimination from the surrounding tissues. Training with the phantom increased the confidence to perform future pericardiocentesis (63 ± 3).

## 4. Discussion

Pericardiocentesis as the standard treatment for pericardial effusion and tamponade has to be performed under great pressure in situations such as shock and resuscitations. In the era of interventional cardiology, the incidence of tamponade has increased and postcardiotomy effusions after hospital discharge can be observed [2, 6].

Echocardiographic guidance has increased the safety and lowered the complication rates [6]. The current ERC guidelines of 2010 enforce the use of ultrasound in peri-resuscitation settings to identify reversible causes such as pericardial tamponade [1].

As most physicians do not practice pericardiocentesis in a controlled environment (i.e., cath lab or ICU) routinely, ultrasound phantoms have been designed as simulation has been shown to increase the competencies of physicians in medical procedures [12].

Available pericardiocentesis trainers are either expensive or nondurable when used multiple times [9, 10]. The goal of our study was to construct a pericardiocentesis phantom that is easily rebuilt, available to small institutions and low-income countries and can be reused multiple times.

The model we constructed costs about 60 euros and can be assembled using readily available materials in about 120 minutes. To reduce further costs, some of the materials can be replaced at an expense of reduced durability.

Zerth et al. published only recently another model but reported that the model can only be stored for 2 weeks at room temperature and used for about 40–50 punctures [11]. As we did use gel wax instead of gelatine, our model will not mould and can be stored for more than two years. Our models were used in average for more than 60 attempted punctures. After those 60 attempted punctures in a training course and 4 months of storage, only the balloon surrounding the celluloid ball had to be replaced as all surrounding structures were still intact (Figure 4(c)). This can easily be done by lifting the silicone skin, replacing the balloon, smoothing out air bubbles, and covering the model with the artificial skin again. Thus, it can be reused with minimum repair effort in discontinuous teaching pathways.

Replacing the gel wax with gelatine may also reduce costs but also will make the model prone to moulding. As a less expensive substitute for the PVC-planks, we used to assemble a square container; any plastic container available in the required format can be utilized (i.e., 2.5 Liters by http://www.becherprofi.at/, product ID 00200835 for 0.81 euro a piece). All of the required materials are readily available, and the construction of the model is independent of specialized tools. As the durability of the presented model in regard to its low cost is high, our development may be used as an alternative to high end simulation equipment. Especially in resource-limited settings, low-cost models are needed for sufficient training. As the model or its components can be transported with minimum space requirements, it can be made available even to remote areas. This is of gaining importance as pericardial effusion and consecutive tamponade, occurring in up to 20%, in developing countries are mostly caused by infectious diseases such as tuberculosis which is regarded as an epidemic in some areas [13].

In contrast to Zerth et al. [11], we performed a standardized evaluation of our model using a questionnaire. Although our pericardiocentesis model was rated only as valuable by the study participants in most regards, it shows clear feasibility and can enrich specialised ultrasound training. However, the lack of a realistic appearance of the phantom itself and the heart was assessed as a weakness. Practicing pericardiocentesis with our model gave the participants more confidence for further interventions whenever in the future they have to perform one.

## 5. Limitations

Our study is limited by the fact that most of the study participants did not have experience with pericardiocentesis and thus could not compare the model to “real life” circumstances. Another limitation is that surprisingly there was no difference between the group of physicians experienced in pericardiocentesis and the group without any experience with punctures of any kind, showing that this can only be of subordinate impact.

## 6. Conclusion

Our low-cost pericardiocentesis model, which can be easily constructed, may serve as an effective training tool of ultrasound-guided pericardiocentesis for acute and critical care physicians.

## Figures and Tables

**Table 1:** List of materials used in our model. Instead of gel wax, gelatine can be used to reduce costs. Any kind of plastic container with a square form is suitable as well.

Material	Retailer (for our model)	Cost (euro)
Container made of PVC-planks, thickness 0.3 mm	Modulor, 10969 Berlin, Art.Nr: 132930	9.50

Gel wax	Mixed Store, 74532 Ilshofen	23.99

Gel (4 l)	Sonosid 1 L, Asid Bonz GmbH, 71083 Herrenberg, PZN 5362311	3.55 (x4)

Balloon about 30 cm	Basis Balloons, Luftballonwelt, 21436 Marschacht, Art.Nr: 90230	0.15

Red ink	Winsor & Newton INK “Deep Red”, England, London HA35RH	4.69

Celluloid ball	Elite 1*, DONIC Schildkröt, D-82515 Wolfratshausen	0.49

Silicon skin/Thera-Band	Schmidt Sports PHYSIO TAPE, 42699 Solingen, Art.Nr. 111202	7.59

**Table 2:** Questions of the evaluation of the phantom. All questions except no. 16 had to be answered via a visual analogue scale (VAS).

No.	Type of question	Question
1	Visualization of structures in short-axis	How well can a pericardium be recognized in the phantom?
2		How well can a heart be recognized in the phantom?

3	Visualization of structures in long-axis	How well can a pericardium be recognized in the phantom?
4		How well can a heart be recognized in the phantom?

5	Visibility and identification of a pericardial effusion	How well can free fluid be seen in the phantom?
6		How well can the fluid be distinguished from the surrounding tissue?

7	Ultrasound-guided puncture	How well is the puncture of the pericardium feasible?
8		How well is the needle tip visible?

9	Aspiration of fluid from the pericardial space	How well is the decrease of fluid visible?
10		How well can a pigtail catheter be inserted?

11	General judgement of the phantom	Is the phantom realistic?
12		How well does the heart correspond to a real heart?
13		Training on this phantom makes me more confident in ultrasound-guided pericardiocentesis.
14		I am experienced in pericardiocentesis…
15		I have already accomplished pericardiocentesis…
16		Profession
